# Adherence patterns in naïve and prevalent use of infliximab and its biosimilar

**DOI:** 10.1186/s41927-022-00295-7

**Published:** 2022-11-01

**Authors:** Chibuike J. Alanaeme, Sujith Sarvesh, Cynthia Y. Li, Sasha Bernatsky, Jeffrey R. Curtis, Huifeng Yun

**Affiliations:** 1 Department of Epidemiology, School of Public Health, University of Alabama at Birmingham, Birmingham, USA; 2 Department of Biology, University of Alabama at Birmingham , Birmingham, USA; 3 Indian Springs School, Pelham, AL USA; 4 Divisions of Rheumatology and Clinical Epidemiology, McGill University Health Centre, Montreal, Canada; 5 Division of Clinical Immunology and Rheumatology, University of Alabama at Birmingham, Birmingham, USA

**Keywords:** Infliximab, Biosimilar, Autoimmune diseases, Adherence

## Abstract

**Introduction:**

Although short-term clinical trials have demonstrated that switching from infliximab (INF) bio-originator to its biosimilar is safe with no significant loss of efficacy, there are limited real-world data comparing their patterns of use and adherence.

**Methods:**

Using 2015–2018 IBM Marketscan data, we established 4 cohorts of patients with at least one administration or pharmacy claim for INF bio-originator or biosimilar in 2017, including INF naïve biosimilar users, INF prevalent biosimilar users, INF naïve bio-originator users, and INF prevalent bio-originator users, defined according to their prior use of INF from 2015 to their first INF administration in 2017. The proportion of days covered (PDC) was calculated for patients with at least 6, 12, or 18 months of follow-up time. Factors associated with optimal adherence (PDC > 80%) were evaluated using log-binomial models.

**Results:**

We identified 96 INF naïve biosimilar users, 223 INF prevalent biosimilar users, 2,149 INF naïve bio-originator users, and 10,970 INF prevalent bio-originator users. At the end of 18 months of follow-up, 64% of INF prevalent bio-originators, 48% of INF naïve biosimilars, 41% of INF naïve bio-originators, and 36% of INF prevalent biosimilars had optimal adherence. Depression, previous hospitalization, and greater use of prior biologics were negatively associated with adherence, whereas IBD diagnoses (referent to RA) and age 55–64 (referent to < 35) were positively associated with high adherence.

**Conclusion:**

INF prevalent users had higher adherence in our analyses than INF naïve users. However, further studies with larger sample size are needed to evaluate INF biosimilar users’ adherence.

## Introduction

Infliximab (INF) is one of the five tumor necrosis factor-α (TNF) inhibitors that is routinely used for indications of chronic inflammatory diseases such as rheumatoid arthritis (RA), inflammatory bowel disease (IBD), ankylosing spondylitis (AS), and psoriatic arthritis (PsA). However, INF treatment can be expensive [1–6], with an estimated annual cost of $21,000 for new initiates, and those continuing therapy paying close to $26,000 per year [2] largely due to dose escalation [1, 3, 4, 6]. Hence, biosimilars were introduced to encourage treatment options and reduce treatment costs through competition [7].

During 2016–2017, the Food and Drug Administration (FDA) approved several INF biosimilars for similar indications as the INF bio-originator, based on shared similarities in the mechanisms of action, routes of administration, dosage form, and strength [8]. These included inflectra (infliximab-dyyb; Celltrion, Inc.) approved in April 2016 [9]; renflexis (infliximab-abda; Samsung Bioepis Co., Ltd.) approved in April 2017 [10]; and ixifi (infliximab-qbtx; Pfizer Inc.) approved in December 2017 [11]. However, optimal adherence, paramount for preventing the associated morbidity and mortality of chronic inflammatory diseases [12–22], is seldom factored in the approval of these drugs.

Sub-optimal adherence (< 80% adherence rate) is common among patients with chronic inflammatory diseases, especially for patients taking biologics. In a Danish study of RA patients switching to biologics after failing disease‐modifying antirheumatic drug (DMARD), adherence was 56% for etanercept, 52% for adalimumab, and 41% for INF. Treatment discontinuation was greater among INF takers regardless of reasons for withdrawal [23]. Similarly, an overall non‐adherence rate of 54% was noticed in a French study for IBD patients continuing INF therapy [24]. Clinical trials have demonstrated that switching from INF to its biosimilar (i.e., inflectra) is safe with no significant loss of efficacy [25]. However, there are limited real-world data that compares their utilization and adherence patterns. Therefore, we compared medication adherence between INF biosimilar and INF bio-originator users and evaluated factors that affected medication adherence using national administrative data.

## Methods

### Data source and study design

We performed a retrospective cohort study using 2015–2018 IBM Marketscan commercial and Medicare claims data, which included de-identified person-level information for over 200 million individuals, encompassing employees, their spouses, and dependents who were covered by employer-sponsored private health insurance or Medicare insurance in the US. These datasets covered enrollment and healthcare utilization across different settings, including demographics, outpatient prescriptions, and diagnostic claims codes for physician office visits, hospital stays, and procedures [26].

### Study cohort

We identified patients with at least one administration or pharmacy claim for INF bio-originator or biosimilar in 2017. We used data from 2015–2017 to classify patients into four groups based on their use of INF before 2017 (index date). These included INF-naïve biosimilar users, prevalent INF biosimilar users, INF-naïve bio-originator users, and prevalent INF bio-originator users. We defined naïve users of INF bio-originator or biosimilar as patients without prior use of INF bio-originator or INF biosimilar before the index date using all available data. Prevalent users of INF bio-originator or biosimilar were defined based on prior exposure to INF bio-originator and no previous use of INF biosimilar. We used 2017 as the index year because we only identified 10 users of INF biosimilar in 2016, of which in 2017, 6 switched to INF bio-originator and the remaining 4 were not defined as naïve biosimilar users. We used the National Drug Code (NDC) and the Healthcare Common Procedure Coding System (HCPCS) to identify claims for INF bio-originator (HCPCS: J1745; NDC: 57894003001) or INF biosimilar (HCPCS; Q5103 and Q5102. NDC; 00069080901 and 32228000101).

To be eligible, patients were also required to be ≥ 18 years of age at the index date and continuously enrolled with full medical and pharmacy health insurance coverage for 2 years preceding their index date (baseline) and through follow up. Follow-up started on the index date and ended on the earliest date of insurance disenrollment, switching from INF bio-originator to INF biosimilar or vice versa or 12/31/2018.

### Assessment of medication adherence

We used the proportion of days covered (PDC) to measure medication adherence rate, which was our primary outcome of interest. The standard dosage schedule for INF bio-originator or biosimilar at time of initiation includes the first administration, and then subsequent treatment at 2 weeks, 6 weeks, and then every 8 weeks subsequently. However, to avoid underestimating adherence, we assigned 8 weeks of medication supply (56 days) per administration [27, 28]. We evaluated the PDC at 6 months, 12 months, and 18 months of follow up. PDC was calculated by dividing each patient’s total days of medication supplied for an interval by the total days of coverage in the interval (183 days, 365 days, and 548 days, respectively).

### Covariates of interest

We included sociodemographic, concurrent medication, and comorbid diseases as potential factors associated with medication adherence. The baseline sociodemographic variables were sex, region (Midwest, Northeast, South, West, and Unknown), and age, which was categorized (< 35, 35–44, 45–54, 55–64, ≥ 65 years) to compare adherence across age groups. We captured past use of biologics (prior to 2017) as at least one NDC or HCPCS code for abatacept, adalimumab, certolizumab, etanercept, anakinra, belimumab, canakinumab, golimumab, ixekizumab, rituximab, sarilumab, secukinumab, tocilizumab, and ustekinumab. To compare adherence between rheumatoid arthritis and other autoimmune diseases, the autoimmune diseases that may have been the underlying indication for INF were hierarchically defined using ICD9/10 codes during baseline. According to order of highest hierarchy, we included rheumatoid arthritis (RA), psoriasis/psoriatic arthritis (PsO/PsA), inflammatory bowel disease (IBD), and others.

Baseline comorbidities were dichotomous and included cancer of any form, chronic kidney disease, chronic obstructive pulmonary disease (COPD), chronic heart disease (CHD), and depression. We identified these baseline autoimmune diseases and comorbidities using physician diagnosed ICD-9 and ICD-10 codes. We included all-cause hospitalization, which was dichotomously defined as any inpatient visit at baseline. Similarly, using a series of ICD9 and ICD 10 diagnoses codes, we identified patients with baseline infections and dichotomously defined them as any inpatient or outpatient infection vs neither. The medications used at baseline that we assessed included steroids, antibiotics, beta-blockers, hormone therapy, opioids, Non-steroidal anti-inflammatory drugs (NSAIDs), and statin. We identified these medications with NDC codes from pharmacy claims.

### Statistical analysis

All variables were categorical and summarized with frequencies and percentages. The chi-square test was used to examine differences in the nominal variables between treatment groups and non-parametric one-way ANOVA (Analysis of variance) was used for ordinal variables. We calculated the PDC of patients with at least 6, 12, or 18 months of follow-up time and categorized their PDC into three groups at these intervals (< 50%, 50–80%, > 80%), choosing conventions commonly used in the literature [29–32]. We defined high adherence as a PDC greater than or equal to 80%, moderate adherence as a PDC of 50–80%, and low adherence as a PDC less than 50% [31].

Log-binomial regression models were used to analyze the baseline characteristics associated with high adherence (i.e., adherence > 80%) adjusting for treatment groups, sociodemographic, number of other biologics at baseline, type of autoimmune disease, comorbidities, baseline medications, and baseline all-cause hospitalization. Both the crude and adjusted relative risks with their 95% confidence intervals were reported. Subgroup analyses were conducted based on patients with prior use of infliximab bio-originator and duration of prior use, and due to sample size, we adjusted for age, number of other biologics at baseline, type of autoimmune disease, and all-cause hospitalization. We reported the relative risks with their 95% confidence intervals. All analyses were conducted with SAS version 9.4 (SAS Institute Inc. Cary, NC).

## Results

**Fig. 1:**
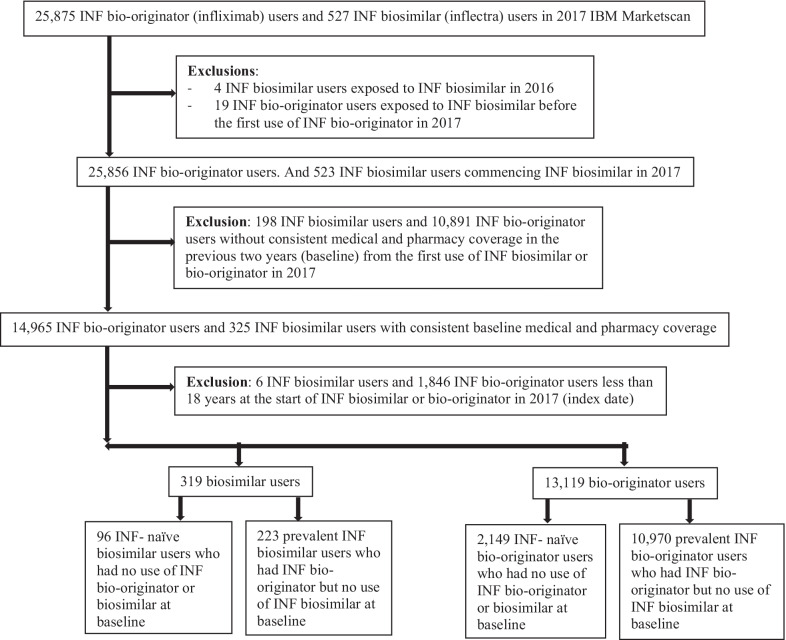
Flow chart for the selection of four INF (infliximab) groups. 96 INF-naïve biosimilar users and 2,149 INF-naïve bio-originator users were ≥ 18 years at index date, had consistent baseline medical and pharmacy coverage and without baseline use of INF bio-originator or biosimilar. 223 prevalent INF biosimilar users and 10,970 prevalent INF bio-originator users were ≥ 18 years at index date, had consistent baseline medical and pharmacy coverage, and had baseline use of INF bio-originator and no baseline use of INF biosimilar

We identified 527 INF biosimilar users and 25,875 INF bio-originator users. After applying the inclusion and exclusion criteria, our final cohort consisted of 13,438 patients, of which 319 were INF biosimilar users and 13,119 were INF bio-originator users (Fig. 1). Among INF biosimilar users, we identified 96 naïve users and 223 prevalent users. Whereas among INF bio-originators, 2,149 were naïve users and 10,970 were prevalent users (Fig. 1).

The baseline characteristics are presented in Table 1. As shown, there were more females than males. Among cohorts, prevalent biosimilar users were older, while naïve bio-originator users were the youngest. Overall, a higher proportion of the INF biosimilar groups were in the west of the United States compared to the bio-originator groups, who were more from the south and the mid-west (Table 1). Regarding autoimmune diseases, the INF biosimilar groups had more RA patients (51%) than the bio-originators (39%), whereas there were more IBD patients in the bio-originator groups (46%) than the biosimilars (36%). In terms of the all-cause hospitalization during baseline, both naïve INF biosimilar (28.1%) and bio-originator groups (28.6%) have higher proportion of patients with baseline hospitalization compared to their corresponding prevalent users. Among these hospitalizations, 7.6% (prevalent biosimilar group) to 23.1% (naïve bio-originator group) of them were due to severe infection, with top four types of infections including abdominal infection, sepsis/bacteremia septicemia, skin and soft issue infection, and pneumonia (data not shown). The proportion of patients with baseline infection was also higher in both naïve INF biosimilar (60.4%) and bio-originator groups (63.9%) than that of prevalent INF biosimilar (54.3%) and bio-originator groups (57.3%). Overall, a larger percentage of the INF-naïve groups had used other biologics at baseline (41%) compared to the prevalent groups (11%).

**Table 1:** Baseline characteristic by treatment group (N = 13,438)

	INF biosimilar (Inflectra)		INF bio-originator (Infliximab)		P-Value
	N = 319		N = 13,119		
	Naïve	Prevalent	Naïve	Prevalent	
Number of patients	96	223	2,149	10,970	
Age, N (%)^a^					< 0.0001*
< 35	24 (25.0)	38 (17.0)	581 (27.0)	2,629 (24.0)	
35–44	14 (14.6)	30 (13.5)	429 (20.0)	1,935 (17.6)	
45–54	21 (21.9)	43 (19.3)	473 (22.0)	2,571 (23.4)	
55–64	27 (28.1)	57 (25.6)	503 (23.4)	2,706 (24.7)	
≥ 65	10 (10.4)	55 (24.7)	163 (7.6)	1,129 (10.3)	
Female, N (%)	63 (65.6)	135 (60.5)	1,330 (61.9)	6,254 (57.0)	0.0001*
Region, N (%) ^a^					< 0.0001*
Mid-West	8 (8.3)	55 (24.7)	402 (18.7)	2,225 (20.3)	
North-East	5 (5.2)	10 (4.5)	351 (16.3)	1,808 (16.5)	
South	32 (33.3)	65 (29.2)	849 (39.5)	4,403 (40.1)	
West	29 (30.2)	71 (31.8)	257 (12.0)	1,318 (12.0)	
Unknown	22 (22.9)	22 (9.9)	290 (13.5)	1,216 (11.1)	
All-cause hospitalization, N (%)	27 (28.1)	39 (17.5)	615 (28.6)	1,466 (13.4)	< 0.0001*
Infections, N (%)	58 (60.4)	121 (54.3)	1,375 (63.9)	6,284 (57.3)	< 0.0001*
Any hospitalized infection during follow-up, N (%)	0 (0.0)	6 (2.7)	108 (5.0)	528 (4.8)	< 0.0001*
Autoimmune disease, N (%) ^a^					< 0.0001*
Rheumatoid arthritis (RA)	51 (53.1)	111 (49.8)	805 (37.5)	4292 (39.1)	
Psoriatic arthritis (PsA)	9 (9.4)	23 (10.3)	125 (5.8)	919 (8.4)	
Inflammatory bowel disease (IBD)	36 (37.5)	78 (35.0)	1,049 (48.8)	4,977 (45.4)	
Others	-	11 (4.9)	170 (7.9)	782 (7.1)	
Comorbidities, N (%)					
Cancer	5 (5.2)	6 (2.7)	83 (3.9)	261 (2.4)	0.0012*
Chronic kidney disease	10 (10.4)	22 (9.9)	210 (9.8)	938 (8.6)	0.2721
Chronic obstructive pulmonary disease	15 (15.6)	19 (8.5)	160 (7.5)	727 (6.6)	0.0087*
Chronic heart disease	4 (4.2)	21 (9.4)	105 (4.9)	505 (4.6)	0.0278*
Depression	14 (14.6)	32 (14.4)	341 (15.9)	1,318 (12.0)	< 0.0001*
Diabetes	9 (9.4)	37 (16.6)	271 (12.6)	1,275 (11.6)	0.0838
Number of other baseline biologics, N (%)					< 0.0001*
None	56 (58.3)	198 (88.8)	1,260 (58.6)	9,777 (89.1)	
One	24 (25.0)	21(9.4)	647 (30.1)	976 (8.9)	
Two or more	16 (16.7)	4 (1.8)	242 (11.3)	217 (2.0)	
Concurrent medications, N (%)					
Antibiotics	70 (72.9)	144 (64.6)	1,572 (73.2)	7,003 (63.8)	< 0.0001*
Betablockers	19 (19.8)	43 (19.3)	309 (14.4)	1,547 (14.1)	0.0839
Hormone therapy	17 (17.7)	37 (16.6)	378 (17.6)	1,608 (14.7)	0.0060*
Opioids	56 (58.3)	115 (51.6)	1,128 (52.5)	4,608 (42.0)	< 0.0001*
Nonsteroidal anti-inflammatory drugs (NSAID)	37 (38.5)	67 (30.0)	660 (30.7)	2801 (25.5)	< 0.0001*
Statin	16 (16.7)	69 (30.9)	399 (18.6)	2206 (20.1)	0.0003*
Steroids	73 (76.0)	75 (33.6)	1,608 (74.8)	4,377 (39.9)	< 0.0001*

Baseline was two years to the index date

* indicates the p-value ≤ 0.05

^a^Estimations were done with one-way ANOVA for ordinal variables and Chi-Square for other variables

**Fig. 2:**
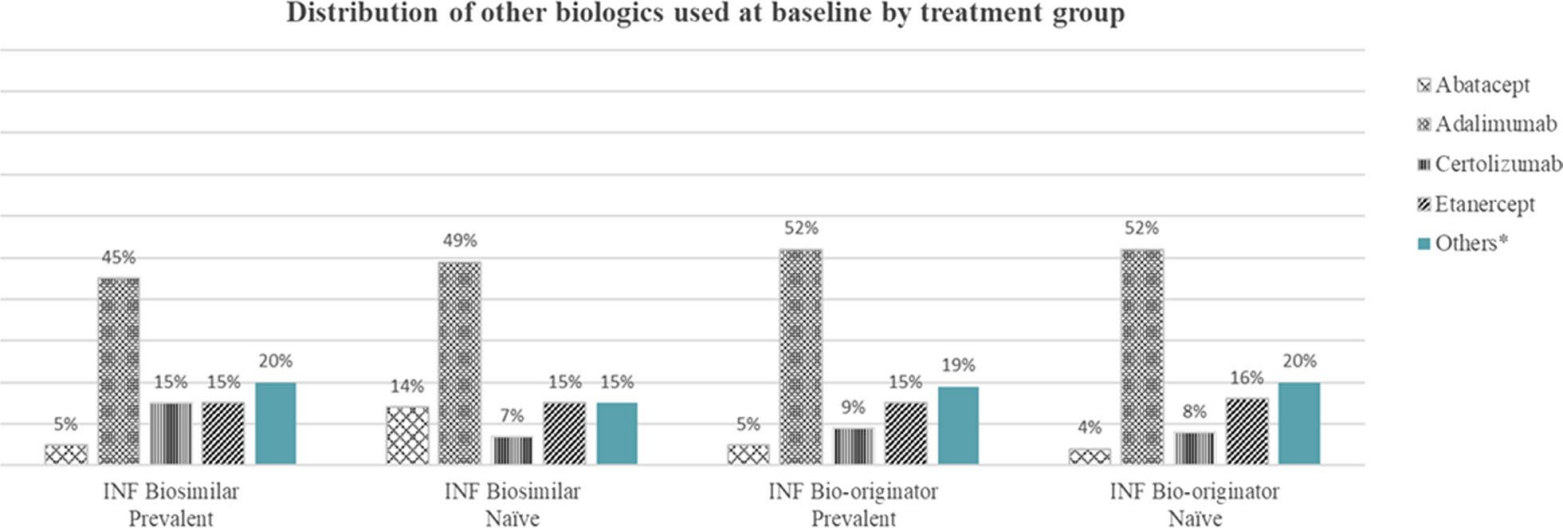
Distribution of other biologics used at baseline excluding infliximab. Adalimumab was more frequently used across groups. *Others included anakinra, belimumab, canakinumab, golimumab, ixekizumab, rituximab, sarilumab, secukinumab, tocilizumab, and ustekinumab

The distribution of baseline biologics (excluding infliximab) is shown in Fig. 2. As shown, adalimumab was more frequently used across groups (45–52%). For naïve biosimilars, the proportion of patients who previously used abatacept (14%) and etanercept (15%) were comparable, with 7% using certolizumab. However, prevalent biosimilar users were equally inclined to use certolizumab (15%) and etanercept (15%) compared to abatacept (5%). Among the INF bio-originator groups, the proportions of patients who used other baseline biologics were similar, with adalimumab being the most frequent, followed by etanercept, certolizumab, and abatacept (Fig. 2).

As the primary outcome, Table 2 shows the adherence rates at three intervals of follow-up (6 months, 12 months, and 18 months), unadjusted for differences in age, sex, or clinical factors. Overall, the proportions of patients with PDC of > 80% decreased in all groups as follow-up progressed. At the 12-month follow-up, prevalent bio-originators had the highest adherence (73% with PDC > 80%), followed by naïve bio-originators (52% with PDC > 80%), prevalent biosimilars (46%), and naïve biosimilar users (43%). At the end of 18 months, prevalent bio-originators still had the highest adherence (64%), whereas the prevalent biosimilars became the lowest (Table 2). However, the adherence for naïve biosimilar users was numerically higher than those of the naïve bio-originator group (48% vs. 41%).

**Table 2:** Adherence at 6, 12, and 18 months of follow-up

	INF Biosimilar (Inflectra)		INF Bio-originator (Infliximab)	
	N = 319		N = 13,119	
	Naïve	Prevalent	Naïve	Prevalent
Number of patient observed	96	223	2,149	10,970
Total person-days	19,008	45, 019	560,142	4,692,189
6—month adherence, N (%)				
< 50%	17 (21.79)	41 (22.78)	250 (13.55)	642 (6.18)
50–80%	14 (17.95)	34 (18.89)	283 (15.34)	1,207 (11.6)
> 80%	47 (60.26)	105 (58.33)	1,312 (71.11)	8,547 (82.21)
Number of patients during follow-up	78 (81.25)	180 (80.72)	1,845 (85.85)	10,396 (94.77)
12—month adherence, N (%)				
< 50%	27 (40.30)	61 (41.50)	485 (30.72)	1,069 (13.46)
50–80%	11 (16.42)	19 (12.93)	276 (17.48)	1,082 (13.62)
> 80%	29 (43.28)	67 (45.58)	818 (51.80)	5,793 (72.92)
Number of patients during follow-up	67 (69.79)	147 (65.91)	1,579 (73.48)	7,944 (72.42)
18—month adherence, N (%)				
< 50%	9 (39.13)	28 (50.00)	350 (42.42)	1,262 (17.06)
50–80%	3 (13.04)	8 (14.29)	135 (16.36)	1,381 (18.67)
> 80%	11 (47.83)	20 (35.71)	340 (41.21)	4,754 (64.27)
Number of patients during follow-up	23 (23.96)	56 (25.11)	825 (38.39)	7,397 (67.43)

Estimated with proportion of days covered (PDC)

Regarding their switching patterns, those who switched to another biologic, or from INF bio-originator to INF biosimilar, or vice versa, were 39% of prevalent biosimilars, 23% of naïve biosimilars, 16% of naïve bio-originators, and13% of prevalent bio-originators. Among these switchers, a large percentage of the INF bio-originator groups and naïve biosimilars switched to another biologic, whereas most of the prevalent biosimilars returned to INF bio-originator (data not shown).

We examined the baseline factors associated with high adherence (Table 3). Among the groups, prevalent bio-originators had higher adherence compared to naïve bio-originators (reference) at all intervals of follow-up (Table 3, 6-months not shown). Prevalent biosimilars were less likely to have high adherence than naïve bio-originators at 12-months (adjusted RR: 0.82 [95% CI 0.68–0.98]). However, there was no significant difference in adherence between the naïve groups in all intervals of follow-up (Table 3). Across age groups, those of ages 55–64 years maintained high adherence as follow-up progressed compared to those younger than 35 years (Table 3). Likewise, IBD patients had better adherence than RA patients throughout follow-up (Table 3). Having depression, any hospitalization, and using other biologics at baseline were negative associates of high adherence.

**Table 3:** Risk ratios for baseline factors potentially associated with high adherence

	12–month adherence (N = 9,737)		18–month adherence (N = 8,301)	
	Crude RR	Adjusted RR ^a^	Crude RR	Adjusted RR ^a^
	[95% CI]	[95% CI]	[95% CI]	[95% CI]
Cohorts				
INF biosimilar naïve	0.83 [0.63–1.10]	0.84 [0.64–1.11]	1.16 [0.75–1.79]	1.21 [0.81–1.82]
INF biosimilar prevalent	0.87 [0.73–1.06]	0.82 [0.68–0.98] *	0.87 [0.60–1.24]	0.76 [0.53–1.08]
INF bio-originator prevalent	1.41 [1.34–1.48] *	1.29 [1.22–1.35] *	1.56 [1.43–1.69] *	1.39 [1.29–1.51] *
INF bio-originator naive	Reference	Reference	Reference	Reference
Age				
35–44	1.05 [1.00–1.09]	1.03 [0.99–1.07]	1.06 [1.00–1.12]	1.04 [0.99–1.09]
45–54	1.08 [1.04–1.12] *	1.04 [1.01–1.08] *	1.11 [1.06–1.17] *	1.05 [1.00–1.09]
55–64	1.10 [1.06–1.15] *	1.08 [1.04–1.13] *	1.18 [1.13–1.24] *	1.11 [1.06–1.16] *
≥ 65	1.02 [0.96–1.08]	0.99 [0.94–1.06]	1.05 [0.98–1.14]	1.00 [0.93–1.07]
< 35	Reference	Reference	Reference	Reference
Sex				
Female	0.96 [0.92–0.97] *	0.99 [0.96–1.02]	0.91 [0.88–0.94] *	0.98 [0.95–1.01]
Male	Reference	Reference	Reference	Reference
Region				
Mid-west	1.02 [0.98–1.05]	1.00 [0.97–1.04]	1.04 [1.00–1.08]	1.03 [1.00–1.07]
North-east	1.02 [0.98–1.05]	1.01 [0.98–1.04]	0.99 [0.95–1.03]	0.99 [0.95–1.03]
West	0.97 [0.93–1.00]	0.98 [0.94–1.01]	0.95 [0.90–0.99] *	0.96 [0.91–1.00]
Unknown	0.55 [0.51–0.59] *	0.59 [0.54–0.63] *	N/A	N/A
South	Reference	Reference	Reference	Reference
Autoimmune disease				
Rheumatoid arthritis (RA)	Reference	Reference	Reference	Reference
Psoriasis/psoriatic arthritis (PsA)	1.06 [1.01–1.12] *	1.03 [0.98–1.08]	1.06 [0.99–1.13]	1.01 [0.96–1.07]
Inflammatory bowel disease (IBD)	1.07 [1.04–1.11] *	1.07 [1.04–1.10] *	1.11 [1.07–1.15] *	1.09 [1.05–1.13] *
Others	0.98 [0.93–1.04]	0.98 [0.92–1.03]	0.99 [0.92–1.06]	0.97 [0.90–1.03]
Comorbidities				
Cancer	0.92 [0.84–1.01]	0.99 [0.90–1.08]	1.05 [0.96–1.16]	1.02 [0.94–1.12]
Chronic kidney disease	0.89 [0.84–0.94] *	0.95 [0.90–1.01]	0.90 [0.84–0.96] *	0.99 [0.93–1.05]
Chronic obstructive pulmonary disease	0.87 [0.81–0.92] *	0.97 [0.91–1.03]	0.79 [0.72–0.87] *	0.94 [0.87–1.01]
Chronic heart disease	0.95 [0.88–1.02]	0.98 [0.92–1.06]	0.93 [0.84–1.02]	0.98 [0.89–1.07]
Depression	0.84 [0.80–0.88] *	0.93 [0.89–0.98] *	0.77 [0.72–0.82] *	0.93 [0.88–0.98] *
Diabetes	0.93 [0.88–0.97]	0.99 [0.95–1.04]	0.92 [0.87–0.98] *	1.01 [0.96–1.07]
All cause–hospitalization	0.80 [0.77–0.84] *	0.90 [0.86–0.94] *	0.76 [0.72–0.81] *	0.90 [0.85–0.95] *
Infections	0.95 [0.92–0.97] *	1.02 [0.99–1.04]	0.93 [0.90–0.96] *	1.02 [0.99–1.05]
Used multiple biologics	0.82 [0.78–0.85] *	0.90 [0.87–0.94] *	0.74 [0.69–0.79] *	0.83 [0.78–0.88] *
Concurrent medications				
Antibiotics	0.92 [0.90–0.95] *	0.97 [0.94–1.00]	0.89 [0.87–0.93] *	0.97 [0.94–1.00]
Betablockers	0.99 [0.95–1.03]	1.03 [0.99–1.07]	0.92 [0.87–0.97] *	1.00 [0.95–1.05]
Hormone Therapy	0.97 [0.94–1.01]	1.01 [0.973–1.05]	1.00 [0.95–1.04]	1.04 [1.00–1.09]
Narcotic	0.91 [0.89–0.94] *	1.02 [0.99–1.04]	0.84 [0.81–0.87] *	0.99 [0.96–1.03]
NSAID	0.94 [0.91–0.97] *	1.02 [0.99–1.05]	0.89 [0.85–0.92] *	1.03 [0.99–1.07]
Statin	0.99 [0.96–1.03]	1.00 [0.97–1.04]	0.97 [0.93–1.02]	0.99 [0.95–1.03]
Steroids	0.89 [0.86–0.91] *	0.99 [0.96–1.01]	0.85 [0.82–0.88] *	0.97 [0.94–1.00]

Estimated with Log-binomial regression

High adherence was defined as adherence rate > 80%

* indicates the p-value ≤ 0.05

^a^Adjusted for age group, sex, region, autoimmune diseases, comorbidities, all-cause hospitalization, number of biologics and concurrent medications. RR: relative risks

In a subgroup analysis, we examined high adherence between the prevalent groups by the length of previous use of INF bio-originator, adjusting for age, type of autoimmune disease, baseline all-cause hospitalization, and number of other biologics at baseline (Table 4). Among the prevalent bio-originator group, those who previously used INF bio-originator for less than 12 months were less likely to have high adherence compared to those whose previous use were ≥ 12 months (Table 4). However, in the prevalent biosimilar group, there was no significant difference in adherence by the length of previous use of INF bio-originator (Table 4). Similarly, there was no significant difference in adherence between the prevalent groups who previously used INF bio-originator for less than 12 months (Table 4). However, in those who previously used INF bio-originator for ≥ 12 months, the biosimilar group were less likely to have high adherence compared to the bio-originator group (Table 4).

**Table 4:** Evaluation of high adherence among prevalent users by length of previous INF bio-originator use in three follow-up intervals

	6 Months^a^	12 Months^a^	18 Months^a^
Among INF bio-originator prevalent users			
< 12 months prior use of INF bio-originator	0.94 [0.92–0.97] *	0.87 [0.83–0.91] *	0.77 [0.73–0.82] *
≥ 12 months prior use of INF bio-originator	Reference	Reference	Reference
Among INF biosimilar prevalent users			
< 12 months prior use of INF bio-originator	0.81 [0.52–1.25]	0.65 [0.35–1.21]	0.64 [0.18–2.35]
≥ 12 months prior use of INF bio-originator	Reference	Reference	Reference
All prevalent INF users with < 12 months prior			
*Use of INF bio-originator*			
INF biosimilar	0.76 [0.53–1.10]	0.61 [0.35–1.06]	0.59 [0.18–1.97]
INF bio-originator	Reference	Reference	Reference
All prevalent users with ≥ 12 months prior			
*Use of INF bio-originator*			
INF biosimilar	0.70 [0.62–0.80] *	0.63 [0.52–0.76] *	0.56 [0.39–0.80] *
INF bio-originator	Reference	Reference	Reference

* indicates the p-value ≤ 0.05

^a^Relative risks were adjusted for age, other biologic use, baseline inflammatory diseases, and hospitalization. Estimated with Log-binomial regression

## Discussion

Multiple factors that affect medication adherence have been reported, including limiting access to health care, using restricted formulary, switching to a different formulary, high cost for drugs, and high copayment [33]. However, the effects of switching from biosimilar to its bio-originators were not well studied in the real-world. Therefore, our study evaluated the adherence patterns among infliximab biosimilar naïve and prevalent users and compared them with its bio-originators.

We performed a retrospective analysis that compared the adherence rate between INF bio-originator and INF biosimilar users and examined the baseline factors potentially associated with high adherence. In all four groups, we found that INF prevalent bio-originators had the highest adherence, and INF prevalent biosimilars had the lowest. Among the baseline factors that might be potentially associated with adherence, we found that patients with depression, previous hospitalization, and using other biologics were less likely to reach optimal adherence, whereas patients who had IBD (reference to RA) and of age group 55–64 (reference to < 35 years old) were positively associated with high adherence.

Our results were consistent with similar studies on adherence to the INF bio-originator used in our work. Kane S.V. et al. reported a 34% non-adherence rate among patients with Crohn’s disease in the first year of infusion-based infliximab therapy [34]. Likewise, Martelli L. et al. found an overall non-adherence of 54% for infliximab among patients with IBD [24]. Also, the adherence rate for infliximab users has been reported as 43% among patients with RA [35]. These studies are consistent with the overall decrease in adherence that we observed.

In our work, we found that IBD patients were positively associated with high adherence than RA patients. This could be due to differences in the presentation of IBD compared to RA. For example, ulcerative colitis (UC), which is a form of IBD and an inflammation of the colon’s mucosa, presents with abdominal pain, hematochezia, and diarrhea [36]. Also, about 33% of UC sufferers experience extraintestinal pain, with arthritis being the most common [36]. However, RA, which is an inflammation of the joints, presents with multiple joint pain and stiffness [36]. A high severity and malaise of IBD over RA could explain the difference in adherence. Another explanation could be the large proportion of IBD patients that we recorded among INF bio-originators compared to INF biosimilars (46% vs. 36%), of which INF bio-originators reported better adherence than INF biosimilars.

Current study also found that all-cause hospitalization was negatively associated with adherence after adjustment. Given that patients with multiple comorbidities and medications were less likely to be adherent and patients with baseline hospitalizations were more likely to have acute conditions and comorbidities that need more medications after discharge, we considered this negative association as consistent with our expectation. We did not find the significant association between baseline infection and adherence, which might have been because we included all inpatient or outpatient infections, and therefore, the infections could simply be due to multiple factors rather than specifically from the use of infliximab.

INF prevalent bio-originators had the highest adherence across cohorts, which was consistent with our expectation. Since these patients used INF bio-originator at baseline and the index date, they were likely to continue due to strong familiarities with their current treatment. Several studies have reported an early higher infection risk to accompany the initiation of biologics [27, 37–39]. Because INF prevalent bio-originators have passed through this early treatment phase where discontinuations due to side effects, tolerability, and lack of efficacy are more common, they were more likely to retain treatment. In fact, INF prevalent bio-originators recorded the least percentage of switchers (13%) compared to other cohorts.

On the other hand, we found that INF prevalent biosimilars users had the largest proportion of switchers (39%), from which most of them returned to the INF bio-originator. A transitory use of INF biosimilar as a substitute for the bio-originator could explain why these users had the least adherence. Among INF prevalent biosimilar users, a strong preference for INF bio-originator could perpetually return some users to the bio-originator. Also, within these users, similar therapeutic effects between INF bio-originator and biosimilar could cause some to alternate between these treatments. Since we censored patients if there was a switch between the medications of interest, we did not capture the degree of alternation or switching from INF bio-originator to biosimilar, and vice versa. However, this is the focus of an ongoing analysis.

In our work, patients from the west reported lower adherence compared to those from the south, although the confidence interval included the null. In contrast, optimal adherence was virtually the same for patients in the mid-west and the south. Geographical differences in physician practice and marketing strategies may be reasons for this phenomenon. Due to the small sample size of individuals who used more than one biologic at baseline, we compared adherence between those who used other biologics at baseline versus those who did not. We found that using another biologic at baseline was negatively associated with optimal adherence, which could be due to a habit of switching among these users. Since these patients were already switchers at baseline, they had an increased tendency of switching during follow-up.

Since several studies have found depression associated with non-adherence [40–44], we expected to see it negatively impact medication adherence. Non-adherence has also been associated with higher odds of previous hospitalizations [35], which we also found in our study. Adherence has been shown to increase with age [45, 46], with those younger than 50 years being more likely to report poor adherence [47]. Advanced age also has a negative effect on adherence due to the accompanied age-related comorbidities like cognitive impairment and physical difficulties [45, 48, 49]. Like these studies [48, 50, 51], we found individuals of ages 55–64 years were more likely to have higher adherence.

### Strengths and limitations

PDC has been used to study adherence to a class of treatment [48, 52–54] and has been shown to provide a conservative estimate of adherence than the medication possession ratio (MPR), especially when patients are likely to switch medications within a class or simultaneously use multiple drugs in a class [52]. Our use of national administrative data ensured geographical representation, with large sample size. And we were also among the first handful of studies to compare medication adherence between an INF bio-originator and its biosimilar. However, our study has several limitations. We assigned 8 weeks of medication exposure (56 days) per administration, which might have over-estimated adherence for the first several months of follow-up. However, the impact of the potential misclassification of exposure for follow-up of 12 and 18 months on adherence was insignificant. Second, we were not able to evaluate dose escalation due to the lack of body weight, absence of drug dose units that was used to count for the strength and unavailable valid algorithms to identify dose escalation based on dose frequency changes, so the residual confounding could exist. Third, the reasons for low adherence were not available, even though we made efforts to adjust for numerous confounders, it does not compensate for imbalance in factors that were not measured and therefore could not be controlled for, such as the prior duration of therapy among the prevalent users and total drug cost. However, in the stratified analysis of adherence between the prevalent groups by the length of previous use of INF bio-originator, the length of previous use of INF bio-originator did not modify adherence between the prevalent groups. In addition, the proportion of patients with hospitalized infections during follow up was less than 5% in all four groups, the potential impact of therapy interruptions should be minimal. Lastly, the small sample size among INF biosimilars (2%) is a limitation that was caused by the recency of its approval and limited uptake in the US.

## Conclusion

In summary, optimal adherence was more common among INF bio-originators, with INF prevalent bio-originators reporting better adherence among cohorts. Among naïve users, naïve bio-originators showed greater adherence, especially in the first 12 months. Further studies with large sample sizes are needed to evaluate the adherence of INF biosimilar users. However, we found that non-adherence was still common in patients with autoimmune diseases, which is a hindrance to preventing the complications accompanying the long-term management of chronic inflammatory diseases. Our future work is examining the real-life health outcomes between INF bio-originators and biosimilars.

## Data Availability

The datasets generated and/or analyzed during the current study are not publicly available due to the data use agreement but are available from the corresponding author on reasonable request.

## Abbreviations

ANOVA: Analysis of variance
AS: Ankylosing spondylitis
CHD: Chronic heart disease
COPD: Chronic obstructive pulmonary disease
DMARD: Disease modifying antirheumatic drug
FDA: Food and drug administration
HCPCS: Healthcare common procedure coding system
IBD: Inflammatory bowel disease
IBM: International business machines corporation
ICD: International classification of disease
INF: Infliximab
MPR: Medication possession ratio
NDC: National drug code
NSAIDs: Non-steroidal anti-inflammatory drugs
PDC: Proportion of days covered
PsA: Psoriatic arthritis
PsO: Psoriasis
RA: Rheumatoid arthritis
TNF: Tumor necrosis factor
UC: Ulcerative colitis
